# Genetic diversity, genetic structure and demographic history of *Cycas simplicipinna* (Cycadaceae) assessed by DNA sequences and SSR markers

**DOI:** 10.1186/1471-2229-14-187

**Published:** 2014-07-12

**Authors:** Xiuyan Feng, Yuehua Wang, Xun Gong

**Affiliations:** 1 Key Laboratory for Plant Diversity and Biogeography of East Asia, Kunming Institute of Botany, Chinese Academy of Sciences, Kunming, China; 2 University of Chinese Academy of Sciences, Beijing, China; 3 Plant Science Institute, School of Life Sciences, Yunnan University, Kunming, China

**Keywords:** *Cycas simplicipinna*, Pleistocene, Genetic differentiation, Population contraction, *In situ*, *Ex situ* conservation

## Abstract

**Background:**

*Cycas simplicipinna* (T. Smitinand) K. Hill. (Cycadaceae) is an endangered species in China. There were seven populations and 118 individuals that we could collect were genotyped in this study. Here, we assessed the genetic diversity, genetic structure and demographic history of this species.

**Results:**

Analyses of data of DNA sequences (two maternally inherited intergenic spacers of chloroplast, cpDNA and one biparentally inherited internal transcribed spacer region ITS4-ITS5, nrDNA) and sixteen microsatellite loci (SSR) were conducted in the species. Of the 118 samples, 86 individuals from the seven populations were used for DNA sequencing and 115 individuals from six populations were used for the microsatellite study. We found high genetic diversity at the species level, low genetic diversity within each of the seven populations and high genetic differentiation among the populations. There was a clear genetic structure within populations of *C. simplicipinna*. A demographic history inferred from DNA sequencing data indicates that *C. simplicipinna* experienced a recent population contraction without retreating to a common refugium during the last glacial period. The results derived from SSR data also showed that *C. simplicipinna* underwent past effective population contraction, likely during the Pleistocene.

**Conclusions:**

Some genetic features of *C. simplicipinna* such as having high genetic differentiation among the populations, a clear genetic structure and a recent population contraction could provide guidelines for protecting this endangered species from extinction. Furthermore, the genetic features with population dynamics of the species in our study would help provide insights and guidelines for protecting other endangered species effectively.

## Background

Historical processes leave imprints on the genetic structure of existing populations, especially those of long-lived and sessile organisms. The present genetic structure of many species has therefore been used to estimate the relationship between historical vicariance and geological change [1], dispersal history [2] and episodes of expansion and contraction associated with global climate change [3]. Climate can influence genetic variation by controlling the demography of a species [4]. The influence of Quaternary climate change on present patterns of genetic variation of some species has been studied [5,6]. Gugger [7] verified that late Quaternary glacial cycles played an important role in shaping the genetic structure and diversity of the present population of *Quercus lobata* Nee. The results showed that *Quercus lobata* maintained a stable distribution with local migration from the last interglacial period (~120 ka) through the Last Glacial Maximum (~21 ka, LGM) to the present. This contrasts with large-scale range shifts in *Quercus alba* L [7]. More recent climatic oscillations have had profound effects on the dynamics of population expansion and contraction, causing populations to contract into glacial refugia, become extinct and possibly to adapt locally [8,9]. Cycads are an ancient plant form, and their current genetic structure and population dynamic history are not fully understood. Therefore, they are valuable for contemporary researchers to study what they experienced in history and how they respond to historic climate change.

Cycads are the most primitive living seed plants. Fossil evidence shows that cycads originated approximately 275–300 million years ago [10,11]. Molecular evidence also shows that cycads originated much earlier than flowering plants [12,13], which originated approximately 125 million years ago [14,15]. Although cycads are generally long-lived [16,17], they presently comprise a relatively small group with two families (Cycadaceae, Zamiaceae) and ten genera [18]. They are currently considered to be the most threatened groups of organisms on the planet [19]. Cycads are distributed in Africa, Asia, Australia and South and Central America; 62% of the known cycad species are threatened with extinction [19]. There is only one cycad genus, *Cycas*, in China, and it is considered to be the oldest cycads genus [20]. All cycads have been given ‘First Grade’ conservation status in China [21].

*Cycas simplicipinna* (T. Smitinand) K. Hill was formally described in 1995. It is distinguishable by having the morphological characteristics of a shrub, an unremarkable trunk, and lanceolate cataphylls and is distributed in the Yunnan Province of China, Laos, Northern Thailand, and Vietnam. The species is dioecious and allogamous. Their seeds are mainly distributed by weight and usually distribute around the mother plant. So the phenomenon of severe inbreeding is common in the species, resulting in the expected high genetic differentiation and structure by using maternally inherited DNA. Despite being a national key protected plant, the genetic diversity and genetic structure of *C. simplicipinna* has not been studied in detail. The reasons for its endangerment are unclear. This study was undertaken to provide better understanding of the species’ genetic diversity and genetic structure and the reasons for its endangerment. Field surveys showed that there are two populations with fewer than 20 individuals. It is urgent to develop effective protection measures that are based on a comprehensive study of its genetic diversity and population structure.

The organelle DNA of cycads is maternally inherited and is dispersed only in seeds [22]. Their nuclear DNA (nDNA) is biparentally inherited and is dispersed by both seeds and pollen. Microsatellite markers (SSRs) are known to be codominant and to have more genetic variation than other molecular markers. In this study, we used cpDNA (*psb*A-*trn*H and *trn*L-*trn*F), nrDNA (ITS4-ITS5) and SSR markers. The main aim of the study was to evaluate the genetic diversity, genetic structure and demographic history of *C. simplicipinna* and to provide basic guidelines for its conservation.

## Methods

### Study species

**Figure 1:**
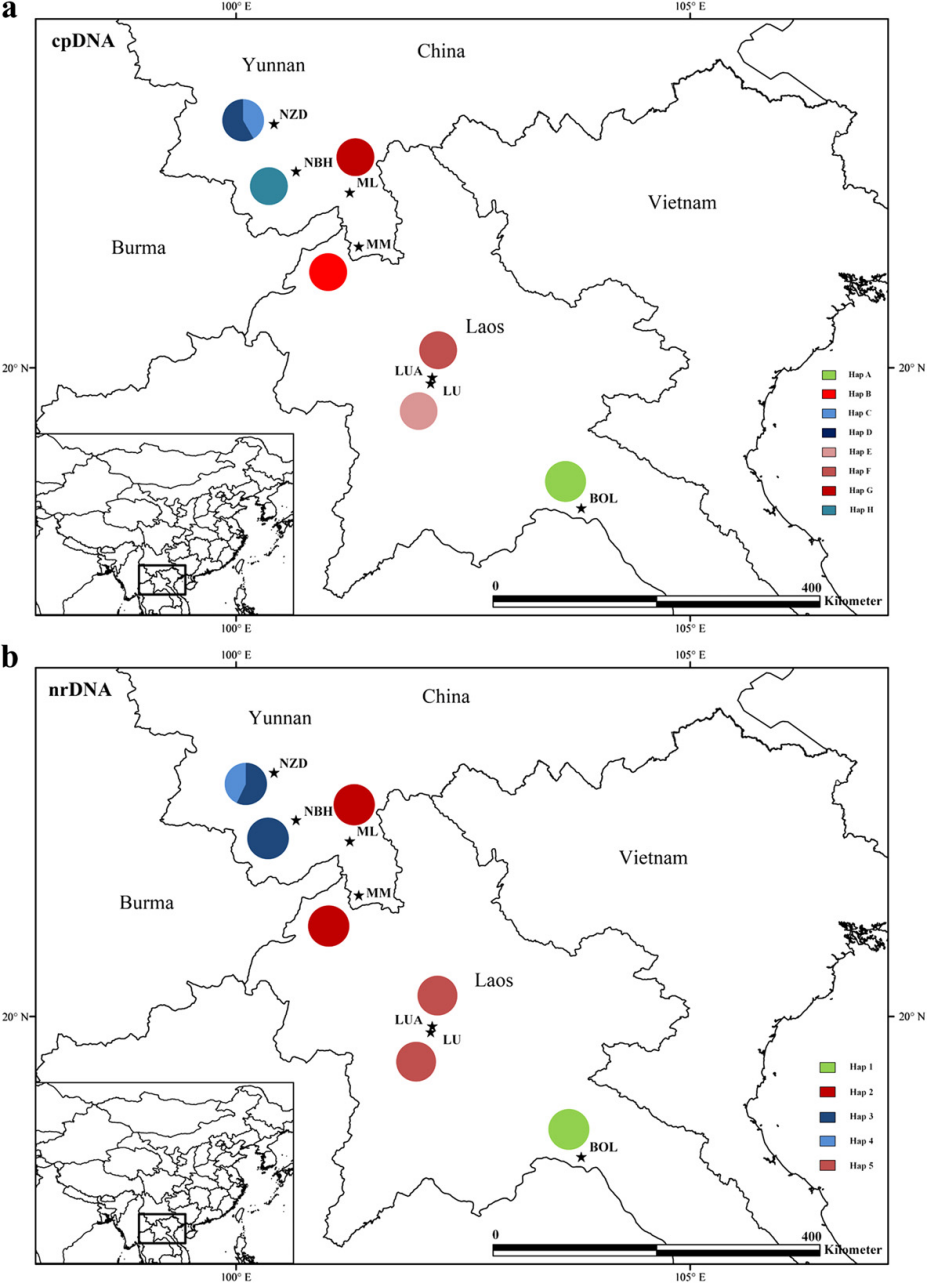
**Distribution of cpDNA (a) and nrDNA (b) haplotypes detected among seven populations of *C. simplicipinna*.** Full names of the abbreviations for the populations are shown in Table 1.

A total of 118 individual samples were collected from seven populations of *C. simplicipinna* (four populations were sampled in Yunnan Province, China and three populations were sampled in Laos). Of the 118 samples, 86 individuals from the seven populations were used for chloroplast and nuclear DNA sequencing. The population known as BOL was eliminated from SSR analysis because there were only 3 individuals. A total of 115 individuals from six populations were used for the microsatellite study. Information on each sampling location and the number of individuals from each population that were used in DNA sequences and SSR analyses are presented in Table 1 and Figure 1, respectively.

**Table 1:** **Details of sample locations, sample sizes (n), haplotype diversity ( *H *** **d) and nucleotide diversity ( *P *** **i) surveyed for cpDNA and nrDNA of *C. simplicipinna***

**Population code**	**Population location**	**Latitude (N°)**	**Longitude (E°)**	**Altitude (m)**	**Individuals for DNA sequences/SSR (n)**	**cpDNA**			**nrDNA**		
						**Haplotypes (No.)**	***H*** **d**	***P*** **i × 10**^**3**^	**Haplotypes (No.)**	***H*** **d**	***P*** **i × 10**^**3**^
BOL	Bolikhamxay, Laos	18.456	103.802	140	3/0	Hap A (3)	0	0	Hap 1 (3)	0	0
LUA	LuangPrabang, Laos	19.897	102.161	300	20/25	Hap F (20)	0	0	Hap 5 (20)	0	0
LU	LuangPrabang, Laos	19.831	102.144	280	20/23	Hap E (20)	0	0	Hap 5 (20)	0	0
MM	Mengman town, Yunnan province	21.128	101.315	640	20/20	Hap B (20)	0	0	Hap 2 (20)	0	0
NZD	Nuozhadu Hydropower Station, Yunnan province	22.690	100.419	780	12/12	Hap C(5) Hap D(7)	0.530	0.37	Hap 3 (12) Hap 4 (9)	0.514	0.95
ML	Menglun town, Yunnan province	21.932	101.253	550	11/11	Hap G (11)	0	0	Hap 2 (20)	0	0
NBH	Nature reserve of Nabanhe, Yunnan province	22.166	100.663	694	10/24	Hap H (10)	0	0	Hap 3 (10)	0	0
Total	7				96/115		0.864	2.59		0.723	8.00

### Molecular procedures

Young and healthy leaves were collected and dried immediately in silica gel for DNA extraction. Genomic DNA was extracted from dried leaves using the modified CTAB method [23]. After preliminary screening of 21–28 samples (representing approximately 3–4 individuals from each population) with universal chloroplast and nuclear primers, we chose two cpDNA intergenic spacers, *psb*A-*trn*H [24] and *trn*L-*trn*F [25], and one nrDNA internal transcribed spacer, ITS4-ITS5 [26], for complete analysis. The three pairs of fragments were amplified for the most polymorphic sites of the 86 individuals. PCR amplification was carried out in 40 μL reactions. For cpDNA, the PCR reactions contained 20 ng DNA, 2.0 μL MgCl_2_ (25 mM), 2.0 μL dNTPs (10 mM), 4.0 μL 10 × PCR buffer, 0.6 μL of each primer, 0.6 μL Taq DNA polymerase (5 U/μL) (Takara, Shiga, Japan) and 26 μL double-distilled water. For nrDNA, the PCR reactions contained 40 ng DNA, 2.4 μL MgCl_2_ (25 mM), 2.0 μL dNTPs (10 mM), 2.0 DMSO, 4.0 μL 10 × PCR buffer, 0.7 μL of each primer, 0.7 μL Taq DNA polymerase (5 U/μL) (Takara, Shiga, Japan) and 24.6 μL double-distilled water. PCR amplifications were performed in a thermocycler under the following conditions: an initial 5 min denaturation at 80°C, followed by 29 cycles of 1 min at 95°C, 1 min annealing at 50°C, and a 1.5 min extension at 65°C, and a final extension for 5 min at 65°C for cpDNA intergenic spacers. For nrDNA sequences we used an initial 4 min denaturation at 94°C, which was followed by 29 cycles of 45 s at 94°C, 1 min annealing at 50°C, and a 1.5 min extension at 72°C, and a final extension for 9 min at 72°C. All PCR products were sequenced in both directions with the same primers for the amplification reactions, using an ABI 3770 automated sequencer at Shanghai Sangon Biological Engineering Technology & Services Company Ltd. For nrDNA, we cloned individuals which had one or more heterozygous sites in the first sequencing round. Six to ten clones were randomly selected and sequenced until the heterozygous site split into two alleles.

Microsatellite markers were selected from recently developed nuclear microsatellites in *Cycas*[27-33]. PCR amplification was carried out in a 20 μL reaction, containing 10 ng DNA, 1.5 μL MgCl_2_ (25 mM), 1 μL dNTPs (10 mM), 1.5 μL 10 × PCR buffer, 0.6 μL of each primer, 0.16 μL Taq DNA polymerase (5 U/μL) (Takara, Shiga, Japan) and 12.14 μL double-distilled water. PCR amplifications were performed in a thermocycler under the following conditions: an initial 4 min denaturation at 94°C, which was followed by 29 cycles of 40 s each at 94°C, 25 s annealing at 48–60°C, and a 30 s extension at 72°C, and a final extension for 10 min at 72°C. PCR products were checked with 8% non-denaturing polyacrylamide gel electrophoresis. Then, we made preliminary screening microsatellite loci for *C. simplicipinna.* The selected microsatellite loci were stained with a fluorescent dye at the 5' end, their PCR products were separated and visualized using an ABI 3770 automated sequencer, and their profiles were read with the GeneMapper software. An individual was declared null (nonamplifying) at a locus and was treated as missing data after two or more amplification failures. Finally, we chose polymorphic microsatellite loci for *C. simplicipinna* after calculating polymorphism indices.

### Data analysis

#### Data analysis of DNA sequences

Sequences were edited and assembled using SeqMan. Multiple alignments of the DNA sequences were performed manually with Clustal X, version 1.83 [34], with subsequent adjustment in Bioedit, version 7.0.4.1 [35]. Two cpDNA regions were combined. A congruency test for the two combined cpDNA regions showed a significant rate of homogeneity (P > 0.5) by PAUP* 4.0b10 [36], suggesting a high degree of homogeneity between the two cpDNA regions. The combined cpDNA sequences were therefore used in the following analysis.

Haplotypes were calculated from aligned DNA sequences by DnaSP, version 5.0 [37]. Within- and among-population genetic diversity were estimated by calculating *Nei’s* nucleotide diversity (*P*i) and haplotype diversity (*H*d) indices using DnaSP, version 5.0 [37]. We calculated within-population gene diversity (*H*_S_), gene diversity in total populations (H_T_ = *H*_S_ + *D*_ST_, *D*_ST_, gene diversity between populations [38]), and two measures of population differentiation, *G*_ST_ and *N*_ST_, according to the methods described by Pons & Petit [39] using the Permut, 1.0 (http://www.pierroton.inra.fr/genetics/labo/Software/Permut). We used the program Arlequin, version 3.11 [40] to conduct an analysis of molecular variance (AMOVA) [41] and to estimate the genetic variation that was assigned within and among populations.

Phylogenetic relationships among cpDNA and nrDNA haplotypes of *C. simplicipinna* were inferred using maximum parsimony (MP) in PAUP* 4.0b10 [36] and Bayesian methods implemented in MrBayes, version 3.1.2 [42]. *Cycas diannanensis* was used as the outgroup. We used Mega, version 5 [43], to construct a neighbor-joining (NJ) tree that was based on the neighbor-joining method without using an outgroup. The degree of relatedness among cpDNA and among nrDNA haplotypes was also estimated using Network, version 4.2.0.1 [44]. In network analysis, indels were treated as single mutational events.

A well-documented evolutionary rate is needed to estimate coalescent time between lineages within populations. We used the evolutionary rates that had previously been estimated for seed plants to be 1.01 × 10^-9^ and 5.1-7.1 × 10^-9^[45] mutation per site per year for synonymous sites for cpDNA and nDNA, respectively. We used BEAST, version 1.6.1 [46], to estimate the time of divergence by using the HKY model and a strict molecular clock. We also used the BEAST program to create a Bayesian skyline plot with seven steps to infer the historical demography of *C. simplicipinna*. Posterior estimates of the mutation rate and time of divergence were obtained by Markov Chain Monte Carlo (MCMC) analysis. The analysis was run for 10^7^ iterations with a burn-in of 10^6^ under the HKY model and a strict clock. Genealogies and model parameters were sampled every 1,000 iterations. Convergence of parameters and mixing of chains were followed by visual inspection of parameter trend lines and checking of effective sampling size (ESS) values in three pre-runs. The ESS parameter was found to exceed 200, which suggests acceptable mixing and sufficient sampling. Adequate sampling and convergence to the stationary distribution were checked using TRACER, version 1.5 [47]. We used a pairwise mismatch distribution to test for population expansion in DnaSP, version 5.0 [37], to further investigate the demography of the species. The sum-of-squared deviations (SSD) between the observed and expected mismatch distributions were computed, and *P*-values were calculated as the proportion of simulations producing a larger SSD than the observed SSD. Arlequin, version 3.11 [40], was also used to calculate the raggedness index and its significance to quantify the smoothness of the observed mismatch distribution. The signatures of demographic change were examined by neutrality tests, Fu’s *F*_S_[48] to detect departures from population equilibrium. They were calculated using DnaSP, version 5.0 [37].

### Data analysis of SSR markers

**Figure 2:**
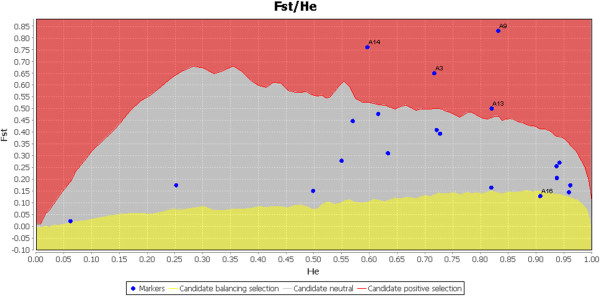
**Test for selection on SSR loci.** Red area represent positive selection, gray area represent neutral selection, and yellow area represent balancing selection. Four loci (A3, A9, A13, A14) subject to positive selection and one locus (A16) subject to balancing selection.

Dataset editing and formatting was performed in GenAlEx, version 6.3 [49]. We tested for evidence of preliminarily selection of our selected loci because our microsatellites had been derived from recently developed nuclear microsatellites of *Cycas*. We also used the *F*st-outlier approach to test for signs of positive and balancing selection on those loci [50,51] by LOSITAN [52]. The outlier loci were identified by the expected distribution of Wright’s inbreeding coefficient *F*st compared with *H*_E_[53]. As recommended by Antao [52], we ran LOSITAN to identify the loci under neutral selection by using the infinite allele model and 10,000 simulations. Twenty microsatellites were first selected after detecting the levels of genetic diversity in the sample of 115 individuals of *C. simplicipinna* in the six populations. The results of positive and balancing selection on the twenty microsatellites detected balancing selection on locus A16 and positive selection on four other loci (A3, A9, A13, and A14). However, locus A13 did not reach the significant level of an *F*st-outlier (Figure 2). Therefore, four loci (A3, A9, A14, and A16) with significant levels as *F*st-outliers were removed from further analysis. Finally, we selected sixteen microsatellites with high polymorphism, stability, and conformity with neutral selection for our research (Additional file 1: Table S1).

The number of alleles (*N*_A_), private alleles (*A*_P_), effective number of alleles (*N*_E_), expected heterozygosity (*H*_E_ = 1-∑*P*i^2^, *P*i, population allele frequencies), observed heterozygosity (*H*_O_ = No. of Hets/N), information index (*I*), and fixation index (*F* = 1-(*H*_O_/*H*_E_)) were calculated using GenAlEx, version 6.3 [49], and POPGENE, version 1.32 [54], with mutual correction. Allelic richness (*A*_R_) was estimated with FSTAT, version 2.9.3 [55], and percentage of polymorphic loci (*PPB*) was calculated with GenAlEx, version 6.3 [49]. Differentiation between pairs of populations was computed using *F*_ST_ and tested with GenAlEx, version 6.3 [49]. Isolation by distance (IBD) was tested on SSR data by computing Mantel tests in GenAlEx, version 6.3 [49] using a correlation of F_ST_/(1-F_ST_) with geographic distance for all pairs of populations. F_ST_/(1-F_ST_) was caculated with Genepop, version 4.1.4 [56]. Gene flow between pairs of populations was estimated using Wright’s principles *N*m = (1-*F*_ST_)/4*F*_ST_[57]. Hardy-Weinberg equilibrium (HWE) was tested for each locus and each population using Genepop, version 4.1.4 [56].

The genetic structures of sampled populations and individuals were estimated by unweighted pair group mean analysis (UPGMA) using TEPGA, version 1.3 [58], with 5,000 of permutations. An individual-based principal coordinate analysis (PCO) was visualized by the program MVSP, version 3.12 [59], using genetic distances among SSR phenotypes. We also conducted a Bayesian analysis of population structure on the SSR data using STRUCTURE, version 2.2 [60]. Ten independent runs were performed for each set, with values of K ranging from 1 to 6, a burn-in of 1 × 10^5^ iterations and 1 × 10^5^ subsequent MCMC steps. The combination of an admixture and a correlated-allele frequencies model was used for the analysis. The second-order rate of change of the log probability of the data with respect to the number of clusters (*Δ*K) was used as an additional estimator of the most likely number of genetic clusters [61]. The best-fit number of grouping was evaluated using *Δ*K by STRUCTURE HARVESTER, version 0.6.8 [62]. Finally, we identified geographical locations where major genetic barriers among populations might occur with a barrier boundary analysis, using BARRIER, version 2.2 [63], based on genetic distance matrices.

We calculated the effective population sizes of each population to establish the degree of endangerment of the species. We used the program LDNe at three levels of the lowest allele frequency (=0.01, 0.02, 0.05) at a 95% confidence interval [64]. We tested the bottleneck statistic at the population level to explore the demographic history of populations by using different models and testing methods implemented in BOTTLENECK, version 1.2.02 [65]. The computation was performed under a stepwise mutation model (SMM) and a two-phased model (TPM). We did not use the standardized differences test in this study because the test was usually used at the condition of having at least twenty polymorphic loci. Two other methods (Sign tests and Wilcoxon tests) were applied to the two models. We also used a mode shift model [66] to test for bottlenecks in each population. These methods implemented in BOTTLENECK have low power unless the decline is greater than 90% [66]. They are most powerful when bottlenecks are severe and recent [67]. In addition, a genetic bottleneck was further investigated with the Garza-Williamsion index (also called *M*-ratio [68], the ratio of number of alleles to range in allele size). When seven or more loci are analyzed, the Garza-Williamsion index is lower than the critical *M*c value of 0.68, a value obtained by simulations based on the empirical data in bottlenecked populations, suggesting a reduction in population size [40,68]. The Garza-Williamsion index is more powerful to detect genetic bottlenecks if the bottleneck lasted several generations or if the population made a rapid demographic recovery [67]. The index was analyzed by Arlequin, version 3.11 [40].

## Results

### DNA sequences

The combined length of cpDNA (*psb*A-*trn*H and *trn*L-*trn*F) varied from 1,408 to 1,438 bp and aligned with a 1,452 bp consensus length that contained 14 polymorphic sites and 16 indels (Additional file 2: Table S2). A total of eight chloroplast haplotypes was identified, and each population was fixed for one particular haplotype, except for population NZD, in which two unique haplotypes was detected (Table 1). The aligned nrDNA (ITS4-ITS5) matrix ranged from 1,079 to 1,087 bp with a consensus length of 1,100 bp that contained 32 polymorphic sites and 11 indels (Additional file 3: Table S3). A total of five nuclear haplotypes was derived. Population BOL had one unique haplotype (Hap 1), MM and ML shared haplotype 2, LUA and LU shared haplotype 5, and NZD had two haplotypes (one was unique and another shared with NBH) (Table 1).

Genetic diversity indices of total nucleotide (*P*i) and haplotype (*H*d) diversity in all populations were, respectively, 0.00259 and 0.864 as inferred from cpDNA and 0.008 and 0.723 as infered from nrDNA (Table 1). Only population NZD showed substantial genetic diversity. Total genetic diversity (*H*_T_ = 1.000, 0.878 from cpDNA and nrDNA, respectively) was higher than the average intrapopulation diversity (*H*_S_ = 0.076, 0.073 from cpDNA and nrDNA, respectively), resulting in high levels of genetic differentiation (*G*_ST_ = 0.924, 0.916; *N*_ST_ = 0.985, 0.992, from cpDNA and nrDNA, respectively Table 2). U tests showed that *N*_ST_ was not significantly greater than *G*_ST_ (P > 0.05) (Table 2), which suggests that there is no correspondence between haplotype similarities and their geographic distribution in *C. simplicipinna*.

**Table 2:** **Genetic diversity, differentiation parameters for the combined cpDNA sequences and nrDNA (ITS4-ITS5) sequences in all populations of *C. simplicipinna***

**Markers**	***H*** **s**	***H***_**T**_	***G***_**ST**_	***N***_**ST**_
cpDNA	0.076	1.000	0.924	0.985
	(0.0758)	(0.0198)	(0.0758)	(0.0166)
ITS4-ITS5	0.073	0.878	0.916	0.992
	(0.0735)	(0.0491)	(0.0828)	(0.0085)

The AMOVA revealed that 98.67% of the genetic variation was partitioned among populations and 1.33% was within populations at the cpDNA level. At the nrDNA level, 97.95% of the genetic variation was partitioned among populations and 2.05% was within populations (Table 3). These results indicate that *C. simplicipinna* has high levels of genetic variation among populations and so high population structure.

**Table 3:** **Analysis of molecular variance (AMOVA) based on cpDNA and nrDNA haplotype frequencies for populations of *C. simplicipinna***

**Markes**	**Source of variation**	**d.f.**	**Sum of squares**	**Variance components**	**Percentage of variation (%)**
cpDNA	Among populations	6	194.292	2.43825	98.67
	Within populations	89	2.917	0.03277	1.33
ITS4-ITS5	Among populations	6	437.219	5.00785	97.95
	Within populations	98	10.286	0.10496	2.05

**Figure 3:**
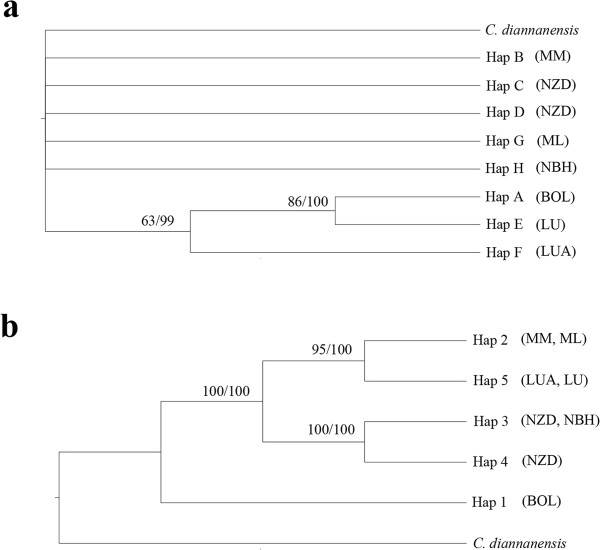
**Strict consensus tree obtained by analysis of eight cpDNA haplotypes (a) and five nrDNA haplotypes (b) of *C. simplicipinna*, with *C. diannanensis *used as the outgroup.** The numbers on branches indicate bootstrap values from the Maximum Parsimony principle (left) and the Bayesian analysis (right). The symbols BOL-NBH in the bracket represent population codes.

**Figure 4:**
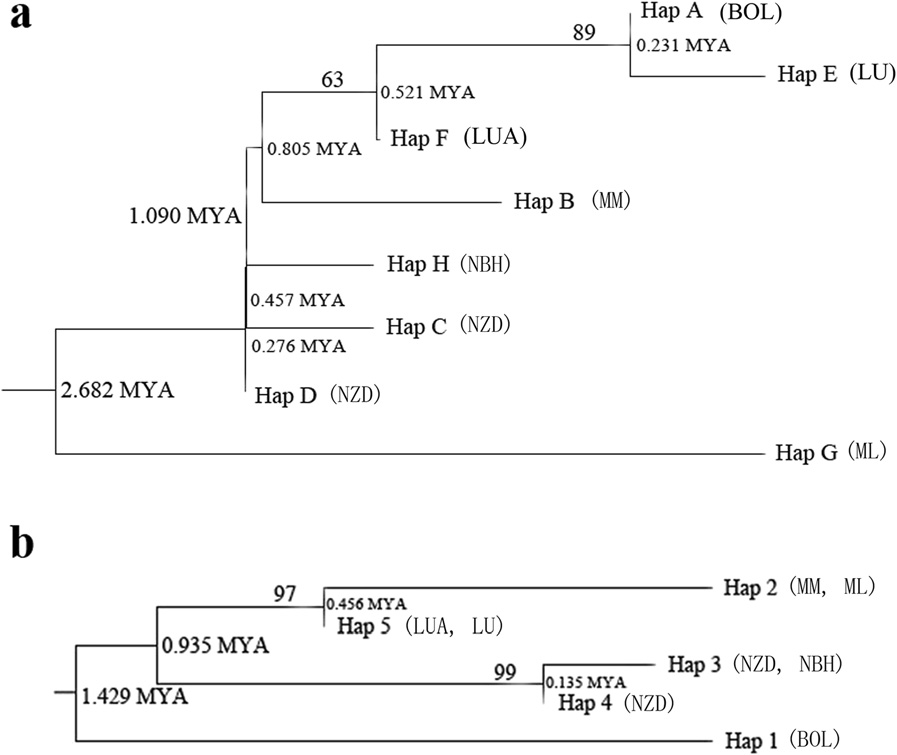
**Neighbor-joining trees were built by using genetic distance based on eight cpDNA (a) and five nrDNA (b) haplotypes of *C. simplicipinna*.** Bootstrap values were shown on branches and divergency times were shown on the nodes. MYA represent million years ago. The symbols BOL-NBH in the bracket represent population codes.

**Figure 5:**
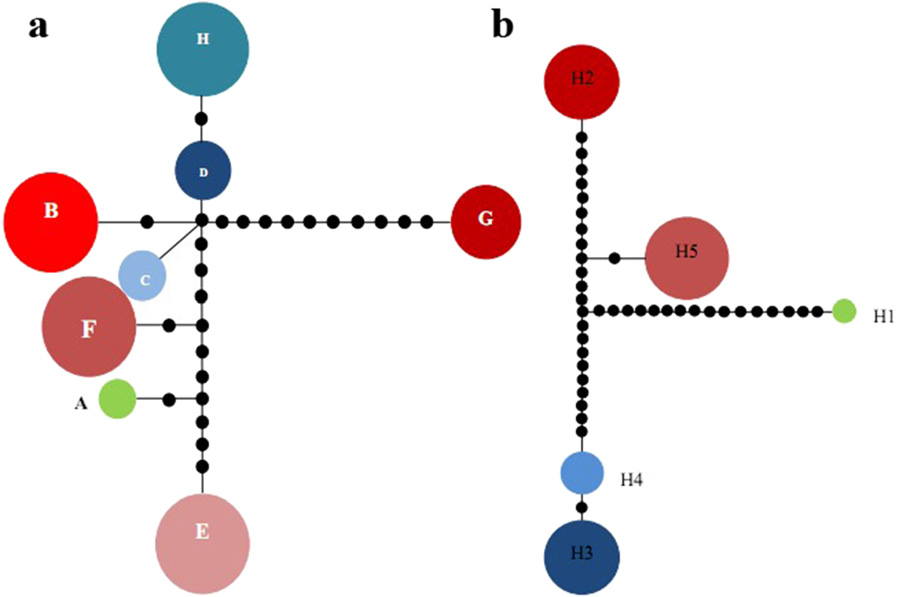
**Network of haplotypes of *C. simplicipinna *based on cpDNA (a) and nrDNA (b).** The size of the circles corresponds to the frequency of each haplotype, the small black circles represents one mutational step.

A phylogeny of cpDNA and nrDNA haplotypes was constructed by both maximum parsimony (MP) and Bayesian methods, using *C. diannanensis* as an outgroup. Both analyses produced phylogenetic trees with consistent topologies (Figure 3). Eight cpDNA haplotypes appeared as a comb-like structure because they lacked enough information sites (Figure 3, a). Five nrDNA haplotypes were clustered into three clades, showing that Hap 2 is more closely related to Hap 5, and Hap 3 is more closely related to Hap 4 (Figure 3, b). The neighbor-joining trees (NJ) supported the congruent phylogenetic relationship of the cpDNA and nrDNA haplotypes (Figure 4). The haplotype network analysis of cpDNA and nrDNA also yielded the same topological relationships (Figure 5). Most haplotypes were distributed in the outside nodes of the reticulate evolutionary diagram, and many missing haplotypes, specifically between Hap 1 and Hap 2, were evident in the reticulate evolutionary diagram of the nrDNA haplotypes (Figure 5, b).

We derived the estimated time of divergence of *C. simplicipinna* with the Bayesian method, using BEAST, version 1.6.1 [46]. The estimated time of divergence ranged from 0.276 MYA to 2.682 MYA according to the cpDNA data and 0.135 MYA to 1.429 MYA according to the nrDNA data (Figure 4). The cpDNA haplotype G (Hap G) was the earliest to diverge. Its time of divergence was estimated to have been 2.682 MYA. The time of divergence of the clade comprising Hap A, E, F, and B and the clade comprising Hap H, C, and D was 1.090 MYA (Figure 4, a). The phylogenetic tree of nrDNA shows that Hap 1 was the earliest haplotype to diverge. Its time of divergence was 1.429 MYA. The time of divergence between the clade comprising Hap 2 and 5 and the clade comprising Hap 3 and 4 was 0.935 MYA (Figure 4, b). These results imply that the *C. simplicipinna* haplotypes were diverged during the Pleistocene (2.6 Ma to 11 ka).

**Figure 6:**
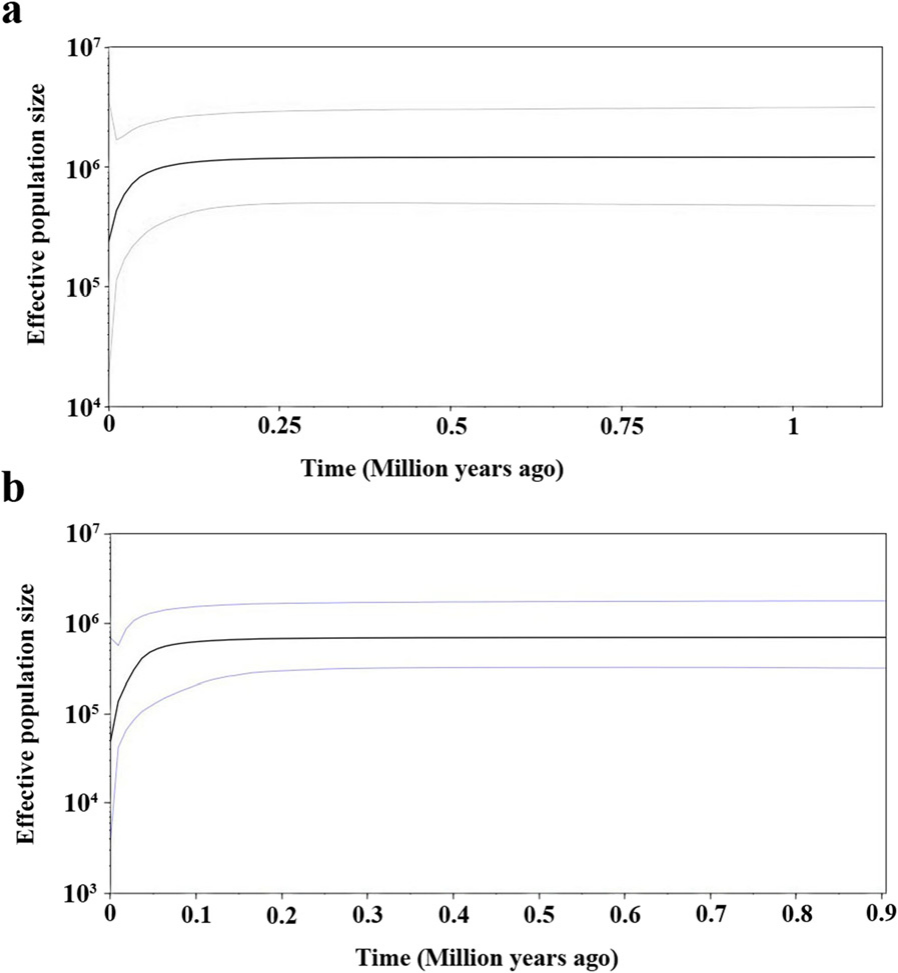
**Bayesian skyline plot based on cpDNA (a) and nrDNA (b) for the effective population size fluctuation throughout time.** Black line: median estimation; area between gray lines: 95% confidence interval.

**Figure 7:**
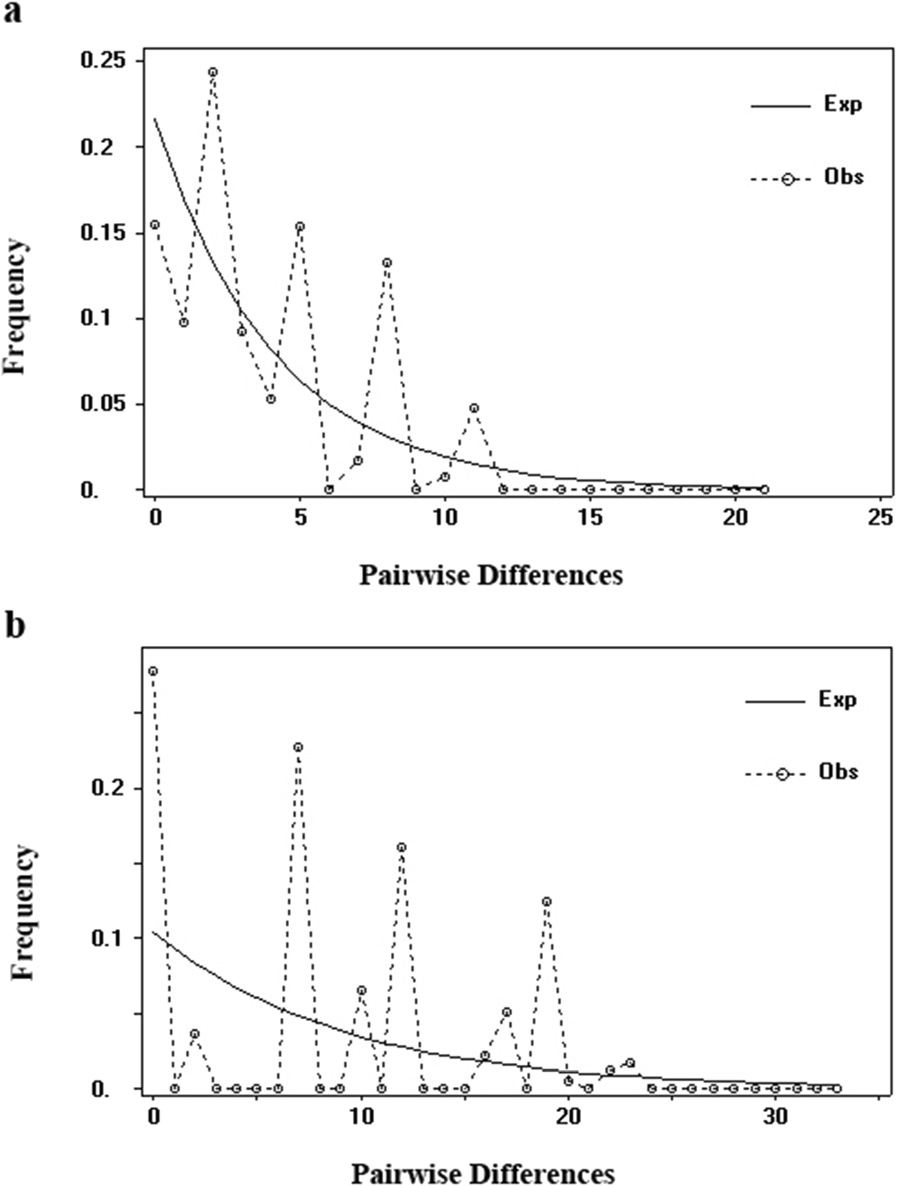
**Mismatch distribution of cpDNA (a) and nrDNA (b) haplotypes based on pairwise sequence difference against the frequency of occurrence for *C. simplicipinna.***

Population dynamic analysis using cpDNA and nrDNA data showed that the population demography of *C. simplicipinna* was stable until approximately 50,000 years ago, at which time a contraction event occurred (Figure 6). The results of the mismatch analysis for all *C. simplicipinna* populations displayed a multimodal distribution pattern (Figure 7) with significant SSD and raggedness values (Table 4), which indicates that *C. simplicipinna* has not undergone a recent population expansion. This conclusion is also supported by the results of the Neutrality Test, Fu’s *F*_*S*_, which yielded positive values (Table 4). Based on a Bayesian simulation, the skyline plot showed recent declines in population size of all populations of *C. simplicipinna* during Quaternary glaciations and no subsequent expansion (Figure 6).

**Table 4:** **Parameters of neutrality tests and mismatch analysis based on cpDNA and nrDNA of *C. simplicipinna***

**Markers**	Fu and Li’ F^*^	**Fu’ *F*** **s**	**SSD**	**Raggedness**
cpDNA	1.570	0.362	0.029^*^	0.112^**^
ITS4-ITS5	2.022^*^	0.439	0.028^*^	0.109^**^

Note: * is *P* < 0.05, significant difference; ** is *P* **< 0.01, the most significant difference.**

### SSR data

**Figure 8:**
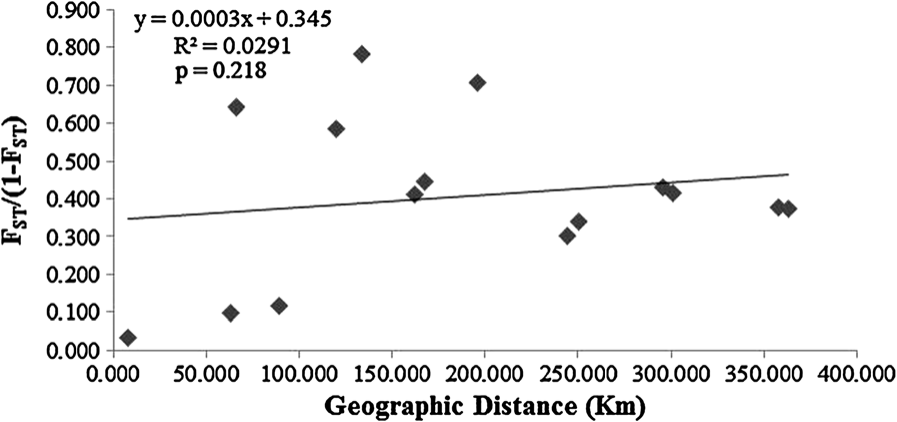
**Plot of geographical distance against genetic distance for six populations of *C. simplicipinna*.**

A total of 169 alleles were identified at the sixteen loci. Diversity estimates varied in different populations (Table 5). Allelic richness was lowest in population MM (*A*_R_, 2.628) and highest in population LUA *(A*_R_, 5.014). The number of alleles (*N*_A_) ranged from 2.875 to 6.063, the number of private alleles (*A*_P_) ranged from 1 to 14, the effective number of alleles (*N*_E_) ranged from 1.925 to 3.521, the information index (*I*) ranged from 0.635 to 1.268, observed heterozygosity (*H*_O_) ranged from 0.306 to 0.473, and expected heterozygosity (*H*_E_) ranged from 0.353 to 0.603. These indices all showed a similar trend, with the lowest values in MM and the highest values in LUA. Fixation indices (*F*) were positive for all six populations, with a mean value *F* = 0.170, which suggests a high level of inbreeding within each population. The percentage of polymorphic loci (*PPB*) was high, ranging from 75% to 100%. Population MM had the lowest genetic diversity, and LUA had the highest. The genetic differentiation coefficient *F*_ST_ varied from 0.036 to 0.467, with a mean value 0.261. No significant effect of isolation by distance (IBD) was detected (Figure 8), as the correlation between genetic and geographic distances was non-significant (P > 0.05), which was supported by the result of Mantel test. Estimates of gene flow between each pair of the six populations are showed in Table 6. Population LUA had the most gene flow with the other populations, and MM had the least. Excesses of homozygotes caused five populations and nine loci to deviate from Hardy-Weinberg equilibrium (Table 5, Additional file 4: Table S4).

**Table 5:** **Genetic diversity and effective population size of six populations of *C. simplicpinna *** **based on sixteen SSR loci**

**Population**	***N***_**T**_	***N***_**P**_	***A***_**R**_	***N***_**A**_	***N***_**E**_	***I***	***H***_**O**_	***H***_**E**_	**F**	**HWE ( *P *)**	***PPB *** **(%)**	**Ne**
LUA	97	14	5.014	6.063	3.521	1.268	0.473	0.603	0.197	0.000^***^	100	164.9
LU	91	6	4.835	5.688	3.402	1.220	0.459	0.589	0.228	0.000^***^	100	33.9
MM	46	1	2.628	2.875	1.925	0.635	0.306	0.353	0.156	0.000^***^	81.25	56.6
ML	53	6	3.312	3.313	2.310	0.773	0.330	0.418	0.190	0.002^**^	75.00	24.8
NZD	64	6	3.889	4.000	2.385	0.885	0.441	0.457	0.093	0.477^ns^	93.75	43.7
NBH	63	8	3.417	3.938	2.292	0.846	0.387	0.442	0.154	0.000^***^	93.75	106.1
Mean	69	6.83	3.849	4.313	2.639	0.938	0.394	0.447	0.170		90.63	71.7

Note: *N*_T_, number of total alleles; *N*_P_, number of private alleles; AR, allelic richness; *N*_A_, number of alleles; *N*_E_, effective number of alleles; *I*, information index; *H*_O_, observed heterozygosity; *H*_E_, expected heterozygosity; F, fixation index; HWE, Hardy-Weinberg equilibrium; *PPB*, percentage of polymorphic loci; Ne, effective population size.-, Monomorphic; *, *P* < 0.05; **, *P* < 0.01; ***, *P* < 0.001.

**Table 6:** **Estimates of gene flow between each pair of the six populations of *C. simplicipinna***

**Population**	**LUA**	**LU**	**MM**	**ML**	**NZD**	**NBH**
LUA	0.000					
LU	6.555	0.000				
MM	0.602	0.558	0.000			
ML	0.816	0.731	2.073	0.000		
NZD	0.653	0.666	0.353	0.425	0.000	
NBH	0.577	0.595	0.319	0.387	2.47	0.000
Mean	1.534	1.518	0.651	0.739	0.761	0.725

**Figure 9:**
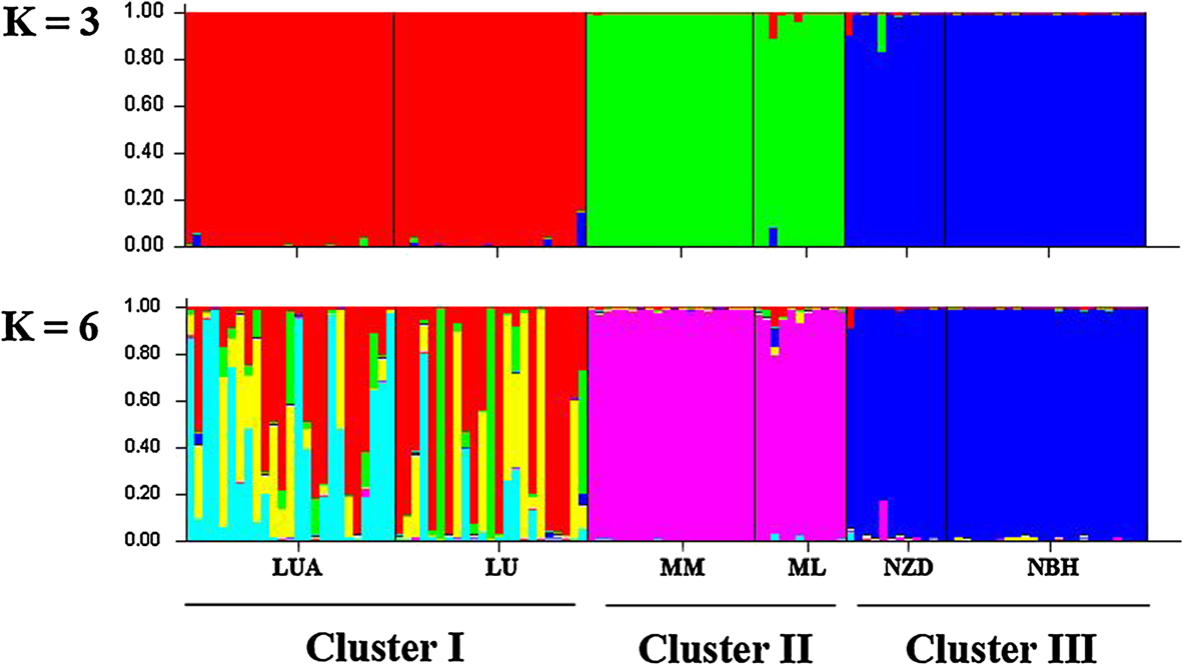
**Estimated genetic clustering (K = 3 and 6) obtained with the STRUCTURE program for six populations of *C. simplicipinna *based on SSR data.** Black lines separate different populations.

**Figure 10:**
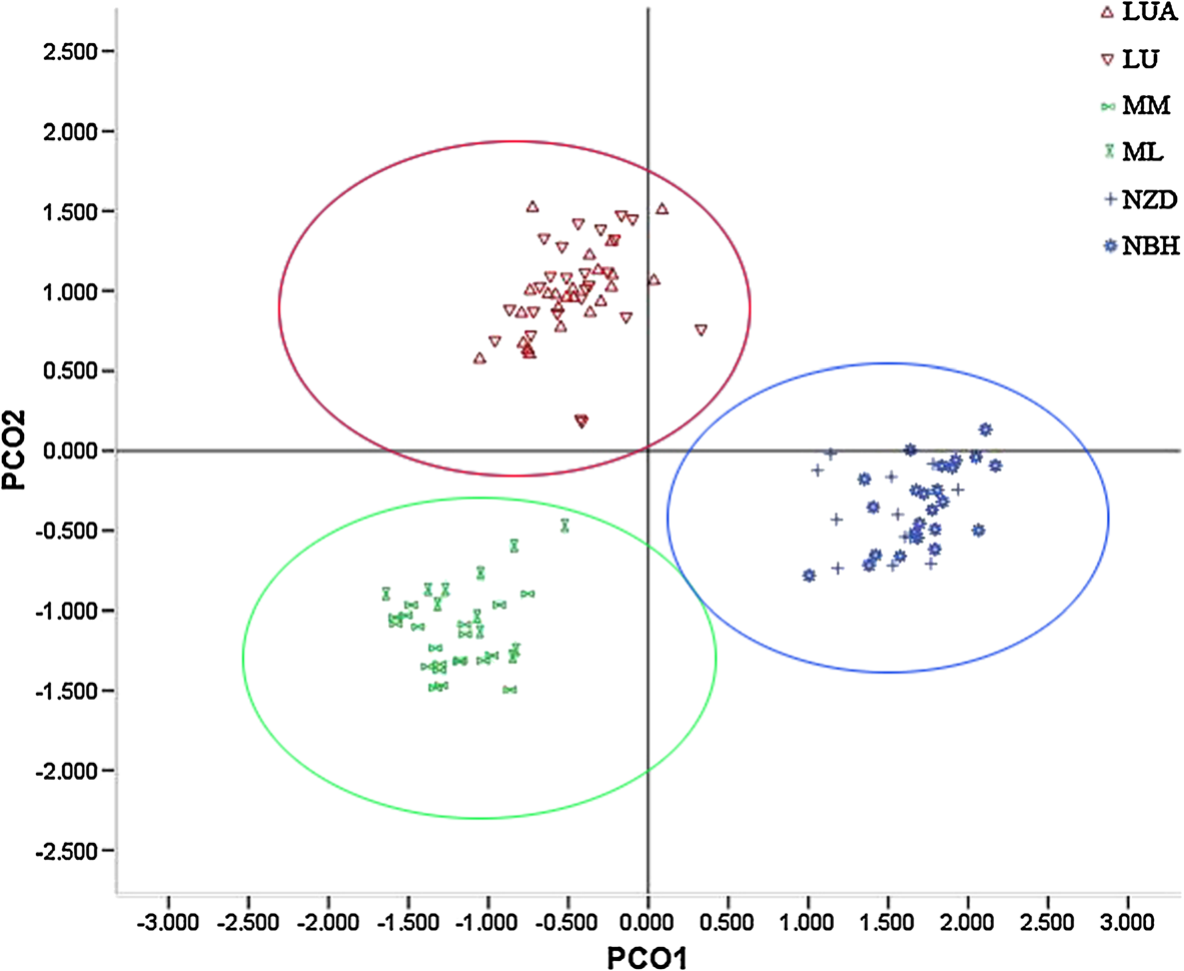
**Principal coordinate analysis (PCO) of SSR phenotypes from six populations and 115 individuals of *C. simplicipinna*.** The symbols LUA-NBH on the figure represent population codes. Colour coding corresponds to the STRUCTURE analysis.

**Figure 11:**
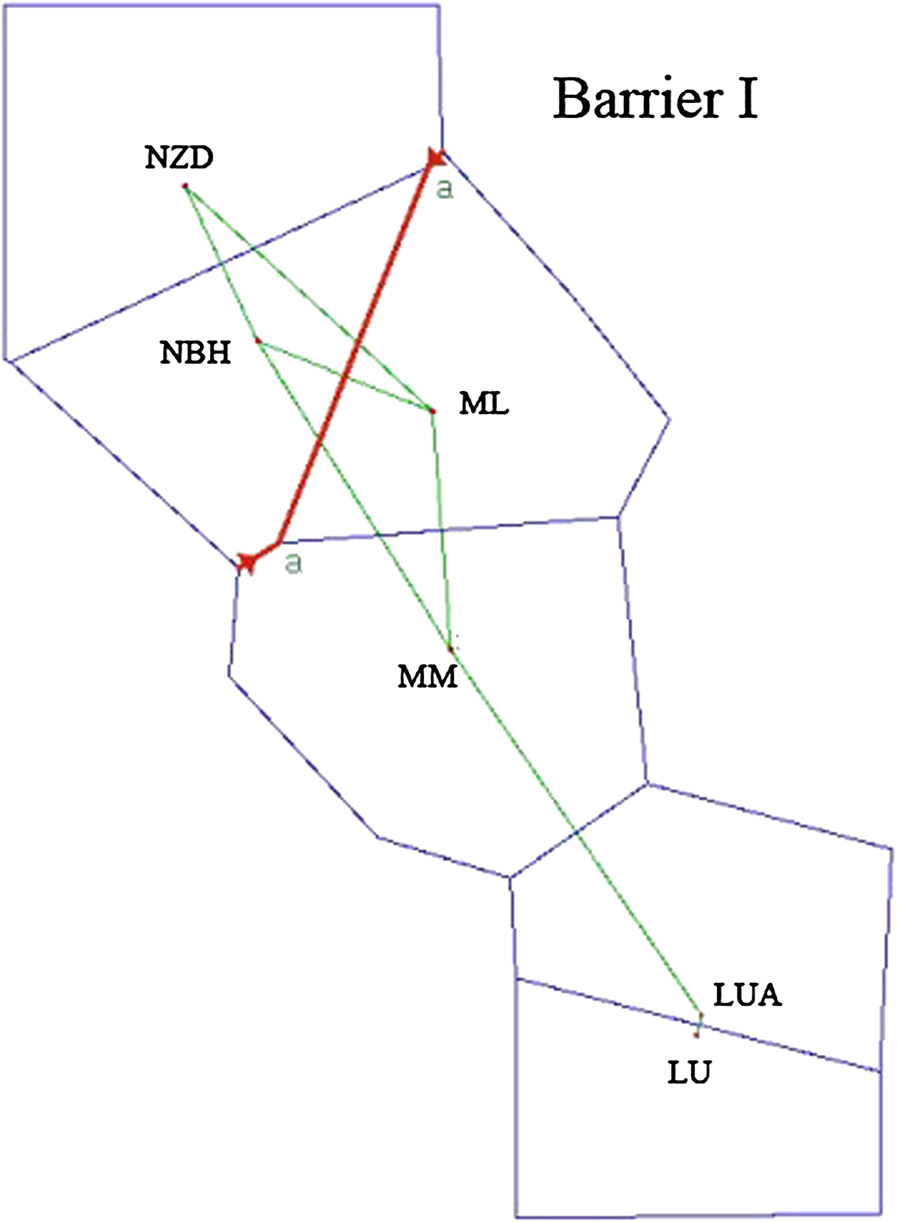
The boundaries detected using the BARRIER program based on matrices of Nei’s (1983) unbiased genetic distance.

The STUCTURE analysis, using the *Δ*K method, showed that the optimal K value was K = 3 (Figure 9), which showed that the six populations were clustered into three groups. Populations LUA and LU were grouped into one cluster (Cluster I), MM and ML were grouped into another cluster (Cluster II), and NZD and NBH were grouped into a third cluster (Cluster III). The result of K = 6 was also present here to detect whether or not has further subdivision in the species. From the Figure 9 we can see that there is only further subdivision at K = 6 between the population LUA and LU. In contrast with K = 6, it is clear that K value was K = 3 is a better solution, because the existence of three groups was also supported by the PCO analysis (Figure 10). Two-dimensional PCO separated all individuals into three clusters along the two axes. The dendrogram (Additional file 5: Figure S1) obtained with the UPGMA clustering method showed that the six populations were separated into three clades with high bootstrap values (100). It is the same as STRUCTURE (K = 3) and PCO analysis. In the UPGMA clustering dendrogram, populations LUA, LU, MM, and ML were clustered into one large clade with a bootstrap value of 78.7. The BARRIER analysis showed that there was only one major genetic boundary (Barrier I), with a 52.7% mean bootstrap value, separating the six populations into two clusters (Figure 11).

Estimates of effective population sizes with the lowest allele frequency (=0.02) as shown by the LDNe analysis are listed in Table 5. The effective population size of LUA and NBH was more than 100 and was less than 50 in three other populations. The BOTTLENECK analysis was used to calculate mutation-drift equilibrium as estimated with different models and different methods (Table 7). This analysis indicates that *C. simplicipinna* did not experience a bottleneck. When TPM was used, only MM had a significant excess of heterozygosity as estimated with the two methods (*P* < 0.05), suggesting that MM deviated from mutation-drift equilibrium. When SMM was used, only ML showed a significant excess of heterozygosity (Wilcoxon text testing). Mode shift models showed that all populations had normal L-shaped distributions, which suggests that *C. simplicipinna* has not experienced a recent severe bottleneck. While all the Garza-Williamson indices (Table 7) of the six populations are lower than the critical *M*c value of 0.68, which indicate that there was a past reduction of effective population size in the species. Populations of *C. simplicipinna* underwent a demographic bottleneck in history.

**Table 7:** **Bottleneck analysis for six populations of *C. simplicipinna *** **under different models and different methods**

**Population**	**T.P.M**		**S.M.M**		**Mode shift**	**Garza-Williamson**
	**Sign test**	**Wilcoxon test**	**Sign test**	**Wilcoxon test**		**index**
LUA	0.156	0.058	0.499	0.430	L	0.466
LU	0.160	0.037^*^	0.497	0.490	L	0.462
MM	0.040^*^	0.008^**^	0.432	0.116	L	0.379
ML	0.294	0.012^*^	0.468	0.047^*^	L	0.349
NZD	0.556	0.470	0.310	0.719	L	0.450
NBH	0.481	0.232	0.333	0.702	L	0.417

Note: *P* is test for heterozigosity excess, *:*P* < 0.05, significant difference; **:*P* < 0.01, most significant difference.

## Discussion

### Genetic variation and genetic structure

The genetic variation of a species is a product of its long-term evolution and represents its evolutionary potential for survival and development [69,70]. Cycads, as ancient gymnosperms with millions of years of evolutionary history, a long life cycle, and overlapping generations, would be expected to have genomes that are responsive to different selective pressures. High levels of genetic variation would be expected to have accumulated during a long evolutionary history. As expected, we found that *C. simplicipinna* has high genetic diversity (Table 1, 2 and 5) at a species level compared with other species of *Cycas* by using similar markers *e.g*., an average value of *H*_T_ = 0.564 and *P*i = 0.00132 were reported for two markers of type cpDNA in *C. debaoensis*[5], and an average value of *H*_O_ = 0.349 and *H*_E_ = 0.545 and the maximum value of *A*p = 2.1, *N*_A_ = 5.8 were reported for 14 markers of type EST-microsatellites in *C. micronesica*[53]. *Cycas simplicipinna* also has higher genetic diversity than many conifers. Many individual conifer species show lower genetic diversity, *e.g*., an average value of *H*_T_ = 0.234 and *H*s = 0.190 were reported for two markers of type cpDNA in *Pinus tabulaeformis*[71], an average value of π = 0.000573 and π = 0.006131 were reported for two markers of type cpDNA and one marker of type nDNA in *Tsuga dumosa*, respectively [72], and an average value of *H*_T_ = 0.77, *H*s = 0.66, *N*_R_ = 3.98, *H*_E_ = 0.62 were reported for seven markers of type nuclear microsatellites in *Taxus baccata*[73]. The mean genetic diversity value of 170 plant species that was estimated from cpDNA-based studies was *H*_T_ = 0.67 [74]. However, at a population level, *C. simplicipinna* shows low genetic diversity; only population NZD has a relatively high genetic diversity.

The genetic diversity of *C. simplicipinna* among all populations (*H*_T_ = 1.000, 0.878 from cpDNA and nrDNA, respectively Table 2) is also higher than the average intra-population diversity (*H*_S_ = 0.076, 0.073 from cpDNA and nrDNA, respectively Table 2), which indicates that there are high levels of genetic differentiation among populations (*G*_ST_ = 0.924, 0.916, *N*_ST_ = 0.985, 0.992 from cpDNA and nrDNA, respectively Table 2). U tests showed that *N*_ST_ was not significantly greater than *G*_ST_, suggesting that there is no distinct phylogeographical structure in *C. simplicipinna*. The *F*_ST_ value of *C. simplicipinna* (nSSR: *F*_ST_ = 0.261, *G*_ST_ = 0.246, Table 5) was higher than the mean value of outcrossing species (*F*_ST_ = 0.22) that was inferred from SSR [75]. Wright [76] had proposed that an *F*_ST_ value greater than 0.25 (*C. simplicipinna*: *F*_ST_ = 0.26 > 0.25) would indicate that there was significant genetic differentiation among populations. Additionally, according to the results of deviation from Hardy-Weinberg equilibrium test (Table 5, Additional file 4: Table S4), only population NZD was in Hardy-Weinberg equilibrium. The remaining five populations deviated significantly from Hardy-Weinberg equilibrium, and the fixation indices (*F*) were greater than zero. We therefore conclude that there is a notable deficit in heterozygosity and severe inbreeding in *C. simplicipinna* populations, resulting in a high among-population genetic differentiation as a whole.

The genetic structure of *C. simplicipinna* based on SSR markers showed that the six study populations were divided into three clades (I, II and III). BARRIER analysis (Figure 11) showed that only one barrier (BS > 50%) exists among the six populations, suggesting that clade I and clade II are genetically more closely related to each other than either is to clade III. Genetic structures of *C. simplicipinna* derived from organelle and nuclear markers are different. It displays high differentiation at cpDNA markers and high distinct structure at biparental markers. This is caused by different features of the markers, such as mutation rates and the biased migration between organelle (pollen migration) and nuclear markers (pollen migration and seed migration, seed migration is very little). Comparisons of genetic with paleoecological data of temperate woody species are known to reveal unique genetic lineages and⁄ or endemic haplotypes in separate refuge populations [77,78]. Although *C. simplicipinna* is a tropical or subtropical woody species, the species similarly possesses unique genetic lineages and endemic cpDNA haplotypes in its separate refuge populations. Duminil [79] had proposed that the level of genetic structure in temperate trees and the potential to reflect historical population isolation are determined in part by life history. Specifically, species with large geographical ranges and wide-ranging seed dispersal display low differentiation at maternally inherited cpDNA markers, and long-lived outcrossing species display low structure at biparental markers. However, *C. simplicipinna* displays the opposite results. This outcome may be due to its having a limited geographic distribution and severe population inbreeding or to its being a tropical or subtropical woody species, which differs from temperate trees.

The correlation between genetic and geographic distances was non-significant (Figure 8), which indicates that there is no significant effect of isolation by distance (IBD). The species lacks of clear geographic structure. It does not automatically mean that the genetic information has no value in directing management. Yang and Meerow [80] estimated that gene flow distance among local populations in *Cycas* was 2–7 km. However, the distances between extant populations of *C. simplicipinna* are all greater than 7 km (the geographic distance between LUA and LU was the smallest of all population pairs (7.52 km)). Because effective gene flow higher than 1 is often regarded as high enough to prevent population differentiation due to genetic drift, while gene flow less than 1 may be a major reason caused the genetic differentiation among populations[81], many gene exchanges between populations less than 1 reflect a low level of gene flow (Table 6). *Cycas simplicipinna* is dioecious and pollenated by insect (Curculionoidea, weevils) [82]. Unlike the birds, weevils can’t spread pollen over a long distance. Its seeds are too large to disperse naturally over such a long distance. Most seeds disperse near the mother plant, which increases inbreeding. Inter-population could not exchange gene flow easily. We therefore conclude that the strong genetic differentiation and structure observed in *C. simplicipinna* is due mainly to its limited gene flow and severe inbreeding. Our analysis therefore suggests that *C. simplicipinna* has high genetic diversity at the species level, low genetic diversity within populations, high genetic differentiation among populations and a clear genetic structure. This conclusion is also supported by the AMOVA analysis (Table 3), which shows that almost all of the genetic variation exists among populations (DNA sequences). Our results support the conclusion that low genetic variation within populations is biologically typical for *Cycas*, unlike other gymnosperms [83].

### Phylogeny and demographic history

Phylogenetic trees constructed on the basis of DNA sequences with different criteria in four different software systems all suggested a consistent systematic relationship of haplotypes (Figure 3 and 4). The comb-like structure of cpDNA haplotypes is likely to be the result of insufficient information site due to insufficient evolutionary time [1,84]. The network analysis (Figure 5) showed that most of the haplotypes were distributed in the outside nodes of the reticulate evolutionary diagrams and there were many missing haplotypes among them. The reason for the observed haplotypes distribution pattern is that diversity within populations is extremely low and differentiation among populations is high. With the exception of the population NZD, the populations have no haplotype and nucleotide polymorphism. This is because that missing haplotypes in the network are due to the species’ long evolutionary history during which climate variations, geological activities, and human activities formed the several scattered populations that currently exist.

Understanding a species’ demographic history aids in understanding its ancient evolutionary environment. Quaternary glaciers are known to have profoundly affected the distribution of plant species [3]. Previous studies have shown that different plant species had different responses to glacial and interglacial influences. Most plant taxa are believed to have shifted the latitude or elevation of their ranges in response to glaciation [8]. Some plant species, *e.g*., *Taxus wallichiana*[85], *C. revoluta* and *C. taitungensis*[6], experienced population expansion during the most recent glacial period, and others, *e.g*., *C. debaoensis*[5], showed population contraction. Although there is growing evidence for population demographic stability or expansion throughout the Last Glacial Maximum (LGM) in a range of different organisms [86-91], *C. simplicipinna* appears to have exhibited population demographic contraction during the LGM and no later expansion. We infer this from the Bayesian skyline plot (Figure 6), a divergence of the observed mismatch distribution from the expected distribution (Figure 7). The significant positive values of SSD and raggedness and non-significant positive Fu’s *F*s (Table 4) also imply *C. simplicipinna* did not undergo a population expansion, so it maybe undergo a population contraction or stay a population dynamic equilibrium. Bottleneck analysis (Table 7) showed that populations of *C. simplicipinna* have not experienced recent bottleneck event but a reduction in population size, which was in accord with Bayesian skyline plot (Figure 6). The divergence times of *C. simplicipinna* haplotypes fall generally in the Pleistocene. We therefore conclude that *C. simplicipinna* was widely and continuously distributed before the glacials and contracted into several isolated surviving populations during the glacials. Refugium populations typically have relatively high genetic diversity and unique haplotypes [77,92]. In our study, *C. simplicipinna* had high genetic diversity at the species level. Each of its population had a unique haplotype for the cpDNA data but low level genetic diversity. The existing *C. simplicipinna* populations are distributed mainly in the tropics or subtropics. Because they are adapted to warm temperatures, temperature is the main factor affecting their growth. The average annual temperature in Chinese subtropical and tropical areas during the last glacial maximum was 4-6°C lower than it is today, which caused change and migration in vegetation [93]. This could have had a strong influence on the distribution of *C. simplicipinna*. The low Ice Age temperatures were not suitable for *C. simplicipinna*, which led to decline or even local extinction of populations. Some cpDNA and nuclear haplotypes were lost in the process, leading us to deduce that the present areas of distribution are its Ice Age glacial refugia. *Cycas simplicipinna* migrated to the scatter refugia that form its haplotypes current distribution pattern. They did not migrate to a common refugium during the Quaternary glacial period but instead survived in their original locales.

We conclude that the reduction in effective population size and limited gene flow were the main factors promoting genetic differentiation among populations of *C. simplicipinna*, which in turn led to the current population structure and distribution pattern.

### Conservation implications

One goal for the conservation of threatened plants is to maintain the genetic diversity of native plant species [94]. *Ex situ* collections are also important because they provide a setting for breeding for introduction, which is an important way to increase the genetic diversity of native plant populations. We found that three (NBH, BOL, ML) of our seven study populations were distributed in protection zones in which there has been no overexploitation and habitat destruction. However, the other four populations (NZD, LU, LUA, and MM) occur outside of protected areas and should be protected. Our study, which revealed a clear population structure of *C. simplicipinna* that show low genetic variation within populations and high genetic differentiation among populations, has significant implications for conservation of this species. The population structure and demographic history of *C. simplicipinna* imply that conservation efforts cannot focus on only one part of the species’ range. We suggest that habitat protection be strengthened immediately by establishing protection zones or plots in the distribution areas of *C. simplicipinna* to improve the conservation awareness of local farmers and to prohibit deforestation in *Cycas* distribution areas. Protection of populations with the highest genetic diversity, such as populations NZD and LUA, should be of highest priority. Priority should also be given to populations with unique haplotypes. Based on the analysis of the two cpDNA sequences in our study, six of the seven sampled populations of *C. simplicipinna* each had one unique cpDNA haplotype. Therefore, every *C. simplicipinna* population should be given maximum protection to prevent losing any haplotypes. The reduction of effective population size in the Ice Age has led to a small effective population size for the species as a whole, which jeopardizes the species’ current genetic diversity. Different populations and/or individuals should be moved from current areas of rich genetic diversity to secure remote areas for *ex situ* conservation. These measures taken together could protect and enrich the genetic diversity of *C. simplicipinna.*

## Conclusions

This study shows that this *cycas* species underwent a past population contraction during Pleistocene with high genetic differentiation among populations and a clear genetic structure. In addition, unique haplotype was detected in all populations in this study. These populations need to be protected for sustaining high genetic diversity in *C. simplicipinna*. Furthermore, the reconstruction of population demographic history in *C. simplicipinna* provides insights and guidelines for protecting *C. simplicipinna* and other endangered *cycas* species effectively.

### Availability of supporting data

The data set of the DNA sequencing data in our study are deposited in GenBank under accession numbers KM065478-KM065496.

## Competing interest

The authors declare that they have no competing interests.

## Authors’ contributions

XG participated in the design of the study as well as collected study materials. XF carried out the molecular genetic studies, participated in the data analysis and drafted the manuscript. YW participated in the study’s design and coordination. All authors read and approved the final manuscript.

## Supplementary Material

Additional file 1: Table S1 Information of sixteen SSR makers used to study the population genetics of *C. simplicipinna*. Click here for file

Additional file 2: Table S2 Variable sites from the two combined cpDNA in *C. simplicipinna*. Click here for file

Additional file 3: Table S3 Variable sites from the nrDNA in *C. simplicipinna*. Click here for file

Additional file 4: Table S4 *P*-value of Hardy-Weinberg equilibrium test for six populations of *C. simplicipinna*. Click here for file

Additional file 5: Figure S1 An unweighted pair-group method with arithmetic averages (UPGMA) phenogram of six populations of *C. simplicipinna* based on SSR markers. Numbers on branches indicated bootstrap values from 5000 replicates. Click here for file
