# Supplementary figures and images for: Genetic diversity, genetic structure and demographic history of Cycas simplicipinna (Cycadaceae) assessed by DNA sequences and SSR markers
Source: BMC Plant Biol. 2014 Jul 12;14:187. doi: 10.1186/1471-2229-14-187 (PMC4114127; doi:10.1186/1471-2229-14-187)

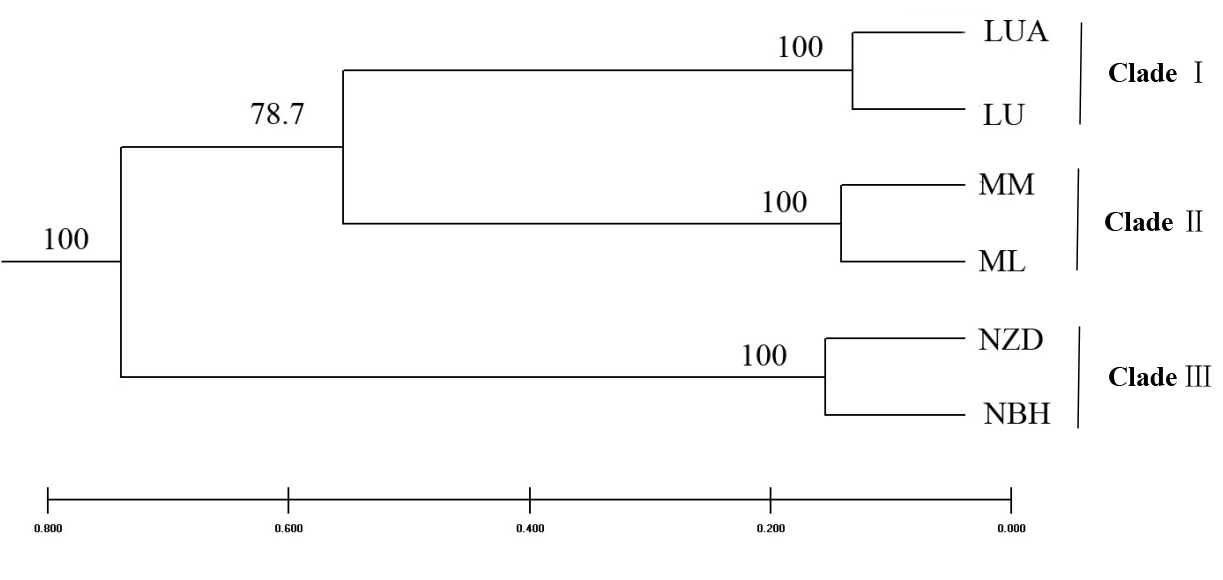

Supplement: Additional file 5: Figure S1 — An unweighted pair-group method with arithmetic averages (UPGMA) phenogram of six populations of C. simplicipinna based on SSR markers. Numbers on branches indicated bootstrap values from 5000 replicates. [file 1471-2229-14-187-S5.jpeg]
